# Places to be young: The dispossession of public space in Old Havana

**DOI:** 10.1177/00420980241249421

**Published:** 2024-05-28

**Authors:** Joanna Kocsis

**Affiliations:** Newcastle University, UK

**Keywords:** displacement, heritage tourism, post-socialist Cuba, touristification, youth, 流离失所, 遗产旅游, 后社会主义古巴, 旅游化, 青年

## Abstract

The touristification of Old Havana is resulting in unique patterns of gentrification that rely on a new spatial imaginary, the enforcement of which is resulting in the loss of places for residents to be young. The Cuban state’s preservation of significant proportions of social housing as part of its investments in the heritage tourism industry is disrupting common housing-led displacement in the city. The neighbourhood’s economic transition is concentrated instead in public spaces, as squares and streets are taken over by new tourist-serving businesses. This process of enclosure dispossesses locals of both public and private leisure spaces, as the cost of consumption in said businesses is beyond the purchasing power afforded by Cuban salaries. The dispossession of public space is particularly problematic for local youth who, given the persistence and pervasiveness of Havana’s housing crisis, spend the majority of their free time in streets and squares. This displacement of youth reinforces existing patterns of exclusion and discrimination along lines of race, class and gender. Given the particular value of public space for youth development in communities like Old Havana, this article documents the three main processes through which young people are being displaced from or dispossessed of urban public space in their neighbourhood, enclosure, sanitisation and temporary appropriation, and discusses the impacts on young peoples’ place-related identity.

## 摘要

哈瓦那旧城的旅游化导致依赖于新空间想象的独特绅士化模式的出现，而这种模式的实施使许多年轻人失去居住的地方。古巴政府保留了大量的社会住房，作为其对遗产旅游业的投资， 此举会中断哈瓦那旧城因住房问题而导致的普遍流离失所现象。随着广场和街道被新的旅游服务企业所占据，街区的经济转型转而开始集中于公共空间。这种圈地过程剥夺了当地人的公共和私人休闲空间，因为上述企业的消费成本超出了古巴工资所能承受的购买力。公共空间的丧失对当地青年来说尤其成问题，由于哈瓦那住房危机的持续和普遍存在，当地青年大部分的空闲时间都花在街道和广场上。青年人的流离失所进一步强化了现存的种族、阶级和性别方面的排斥和歧视。鉴于公共空间对于像哈瓦那旧城这样的社区的年轻人发展具有特殊的价值，本文记录了年轻人被迫离开或被剥夺其街区内城市公共空间的三个主要过程：封闭、消毒和临时占用，并讨论了这些过程对年轻人与地点相关身份的影响。

## Introduction

Touristification (Picard, 2003) tends to involve the modification of space in three discernible ways: a perceivable change in the built environment, a change in the use of existing space or infrastructure and a symbolic or discursive change in the value of a space which can be understood by changing practices in or attitudes towards it (see Freytag and Bauder, 2018 for more). In the case of Old Havana, where such changes are largely taking place in public spaces, transformations in the built environment and its use are leading to symbolic changes that impact the place identity of locals. By prioritising tourism, the tangible spatial practices of enclosure, sanitisation and temporary appropriation communicate messages about the relative worthiness of local people that impact how they see themselves. This spatial–symbolic relationship is particularly relevant in the lives of adolescents, who are deep in the developmental process of identity formation and who spend most of their time in the public spaces being claimed by the tourism industry.

This article identifies how the spatial practices involved in the enactment of Old Havana’s new imaginary dispossess local youth of ‘backstage’ (Goffman, 1978) spaces for the work of identity formation, and change the symbolic value of local youth to the reimagined Old Havana, communicating messages about their worth that impact how they see themselves. It documents how local youth’s experience of the economically and geographically restructured city impacts their imagined futures by centring consumption in ways that can make them feel out of place in their own community. Young people’s identities are tied to the worthiness and entitlement they feel, which, in Old Havana’s new spatial–symbolic dynamic, are conditioned by the relationship that the reimagination of the neighbourhood creates between locals and tourists.

## The socio-spatial development of Old Havana

Old Havana has been the home of low-income communities created by colonial violence for over two centuries, and the neighbourhood’s current transformation threatens to reinforce the economic and social exclusion of these groups. Once the centre of the tangible and symbolic value of the Spanish empire, the colonial walled city of Havana became the neighbourhood of the poor, the previously enslaved and the working class when the physical restrictions imposed by the city walls began to cause overcrowding and inadequate infrastructure (Segre et al., 1997). As wealthy families abandoned their houses, they were replaced by people from informal communities living in ‘*solares, cuarterías, cuidadelas, pasajes* and *accesorias*… dilapidated rooming houses that resulted from the transformation of the old baroque, neoclassical, and eclectic mansions of Habana Vieja’ (Taylor, 2009: 44).

By the late 1800s, the *intramuros* community was no longer the centre of colonial wealth and power, but rather a ghetto to be avoided. Accordingly, the attention and investment meant to create an idealised Havana, a modern capital city of European standards, were concentrated in the *extramuros* area, creating infrastructure to serve the wealthy criollos (people of full Spanish descent born in Cuba) now inhabiting that space (García, 2016) and starving Old Havana of resources. Not coincidentally, Old Havana has had one of the island’s highest concentrations of Afro-Cuban residents since this transition (Zabala Argüelles, 2021). Subsequent governments reinforced the ghettoisation of Old Havana, finding little value in the antiquated and crumbling neighbourhood populated by poor and racialised families. By the time of the Cuban Revolution in the 1950s, housing deficit was one of the major concerns fuelling unrest, but the Revolutionary government embraced an antiurban policy, investing most of its new housing stock in the desperately poor countryside (Scarpaci, 2000). The government did, however, pass the Urban Reform Law of 1960 which expropriated property from urban landlords, redistributed dwellings and established accessible rents that could not exceed 10 per cent of a family’s income (Luzón, 1988; Taylor, 2009). As a result of this law, most residents of *solares* in Old Havana enjoy usufruct rights to their dwellings but cannot sell or easily exchange them. Despite Revolutionary efforts to reduce spatial exclusivity, such reforms to home ownership, and the usufruct system, ‘virtually froze people in neighbourhood space, [and] caused many of the [highly racialised and class-specific] residential patterns that originated in the Republican era to persist’ to the present day (Taylor, 2009: 57).

Upon the dissolution of the Soviet Union, Cuba’s main economic ally, the Office of the Historian of the City of Havana (OHCH) joined legions of city leaders around the world engaged in urban entrepreneurialism (Harvey, 1989) and began a campaign of city marketing (McCann, 2009) designed to remake Old Havana as a heritage tourism destination. As a result, the material relations with space in Old Havana, or the ways in which society procures value from the environment, have once again changed. A place that was once the physical and symbolic fortress protecting the spoils of colonial conquest, and then a residential ghetto abandoned to the poor and working class, has become a site through which economic value is generated via the enclosure and commodification of both space and culture. This change in the material relations with the space of Old Havana has led to a substantial geographical and economic restructuring which has unique impacts for young people whose use of that space is of specific developmental significance.

## Touristification, gentrification and the post-socialist city

With the increasing international mobility of bodies and resources for leisure and labour, concern for the socio-spatial justice of urban transitions is also growing. While the policies of post-Revolutionary Cuba have historically helped the island’s cities to avoid the more severe impacts of touristification and gentrification, recent policy reforms have increased its exposure to such processes, particularly, I argue, in Old Havana.

The impact of heritage tourism on historic city centres in capitalist economies is well documented (Almeida-García et al., 2019; Leccis, 2023; Valverde et al., 2022). Jover and Diaz-Parra (2020) and Diaz-Parra and Hernandez Cordero (2023) describe how processes of exclusion, museumification and commodification affect residents’ access to Lefebvrian rights to a non-alienated urban life and the ability to appropriate neighbourhood space in the historic centre of Seville, Spain, as use values of space are subordinated to exchange values through privatisation and enclosure. The integrity of public space has also been a point of concern in post-socialist urban transitions worldwide. Drummond (2007) and Hykes and Turner (2020) document how the loss of formal public spaces in Hanoi during Vietnam’s economic transition caused young people to use the city differently, as concerns over safety create unequal access to both public spaces and pseudo-public spaces, such as shopping centres, forcing negotiations and compromises regarding which people can use such spaces at which times (Hykes and Turner, 2020: 158). In Eastern Europe and Russia, where public spaces were valued as key sites of political and social connection and personal restoration (Zupan and Büdenbender, 2019), the collapse of socialism drove concerns for public space down the list of policy priorities (Dushkova et al., 2016). As these states adapt to the new demands of emergent socio-economic conditions, market-orientated post-socialist urban planning frameworks are leading to transformations in urban public spaces, often including their degradation or disappearance (Kristiánová, 2016; Vasilevska et al., 2020). The use value of such spaces, especially for young people and children (Nedučin and Krklješ, 2018), is increasingly disregarded, as public spaces are reconceptualised by the state as underutilised spaces for potential commerce or construction (Kalyukin et al., 2015). While the transformation in public spaces in capitalist historic centres, and post-socialist cities in general, is largely driven by the spatial and functional fragmentation in housing (Vasilevska et al., 2020: 83), in Old Havana the preservation of social housing has slowed the process of housing gentrification, while the concentration of investment in the restoration of streets and squares of historic significance has created patterns of displacement and dispossession that disproportionately impact adolescents, and therefore are largely ignored.

## Young peoples’ place(s)

The importance of place in the process of identity formation is increasingly acknowledged by human geographers, who recognise that ‘it is in the transactions between people and their everyday socio-physical environments that identity is created’ (Prince, 2014: 698). Place identity is a substructure of self-identity consisting of positive and negative affects, symbols and beliefs correlating to one’s physical environment (Proshansky, 1978), ranging from belonging to alienation (Prince, 2014). Positive place identity depends on a sense of familiarity or ‘insideness’, an affective evaluation of belongingness (Dixon and Durrheim, 2004). ‘Who I am’ is tied closely to ‘where I belong’.

‘Where I belong’ has as much to do with how I feel about a space as with how the people around me imagine that space should be and view my role in it. Spatial imaginaries, understood here as ontologically performative (Watkins, 2015) with Gregory (2004), are ways of thinking and knowing about places that impact how those places are experienced. Understanding where spatial imaginaries come from, how they are made material and what they are doing is important work for scholars concerned with the ways in which global processes of economic and social restructuring are experienced at the scale of the community. Spatial imaginaries can work to ‘other’ (Sharp, 2009) by creating hierarchies or positive and negative categories of people, places and ways of being. Uncovering spatial imaginaries that have been naturalised can help us to understand the ‘others’ they create (Watkins, 2015: 511). By ‘emplacing’ young peoples’ experiences of identity, we can unpack the relationship between everyday experiences of belonging, spatial imaginaries and the behaviour of youth.

Young people are left out of the reimagination of Cuba through the lens of tourism in ways that leave them vulnerable to exclusion and vilification. While Cuban adults are an important productive component of the tourist industry as the labour that powers tourist services, and Cuban children playing innocently in the streets are a defining element in the aesthetic of idyllic socialist Cuban culture, Cuban teenagers fall between the cracks of these two categories. Cuban teenagers are most often associated with tourism through warnings about *jineteros*, or hustlers, who are also often engaged in sex work with tourists. While in other parts of Latin America young females are associated with sex work while young males are associated with gangs and crime (Oettler, 2011), in Cuba, both sexes are frequently accused as *jineteros* when caught sharing public space with foreigners. While Cubans of all ages are objectified by the tourist gaze through what Collins (2011) labels the commodification of human practice, teenagers are more likely to be seen as risky or extractive (likely to harm or take advantage of tourists) as opposed to productive like adults (who serve tourists materially) and children (who serve the tourists’ imaginary).

There is a rich Cuban scholarship on youth within domestic social work and psychology disciplines, with increasing attention to the impacts of oppression (e.g. Domínguez García, 2010), specifically identifying concerns for the impacts of this current economic crisis on the recreation activities and the identity work of young people (González Herrera and Morales Chuco, 2023; Morales Chuco, 2021). Critical scholarship on the intersections of sex, race and poverty for Cuba’s youth often works through analysis of their art, music and performance (e.g. Fernandes, 2003, 2006; Perry, 2016). From the perspective of urban planning, I add to this important project some details about the accompanying spatial transformations and how they impact young lives.

**Figure 1.**
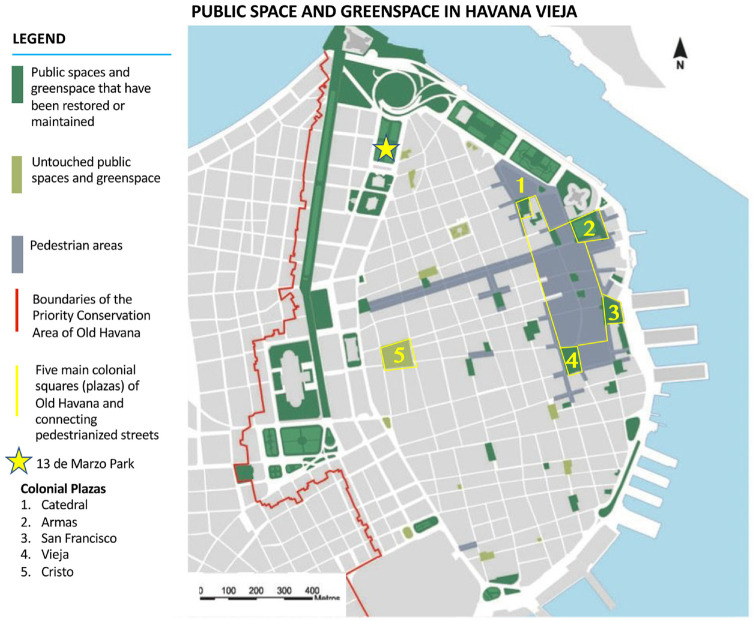
Public space in the priority conservation zone of Havana Vieja. *Source*: Adapted from Rodriguez Alomá and Fornet (2011).

The study discussed here explored the free-time experiences of adolescents living in Old Havana to better understand how and where they used space in their unstructured, unsupervised time for identity work and meaning making. As part of my doctoral research, for eight month-long periods between September 2016 and April 2019 my research assistants^1^ and I spent our evenings with a group of 15 Afro-Cuban teens (five males and 10 females, ages 12–16 when we met^2^) living in dangerously dilapidated usufruct housing in Old Havana. I will herein refer to this group by the term they used to refer to themselves, ‘the muchachos’ (teens). While all of the original housing within Old Havana is overcrowded and in extremely poor condition, restoration efforts are concentrated in the northeast, around the colonial plazas and Malecon (see Figure 1). The muchachos lived in *solares* or apartments within or close to the pedestrianised area or colonial plazas, the part of the neighbourhood that has been prioritised for restoration and has the most heritage tourism traffic. We engaged the muchachos in a range of participatory and arts-based research activities and the research team undertook observant participation (Wilkinson, 2017). Together, we created two popular theatre-style plays and one short film^3^ exploring stories of ‘people like you’. The muchachos took us on guided neighbourhood tours (Gunter, 2010; Loebach and Gilliland, 2010) and created their own neighbourhood maps (Amsden and VanWynsberghe, 2005). They kept personal diaries (Alaszewski, 2006) for several weeks and discussed them with me. We had Cinedebates in which we watched and discussed films about their neighbourhood together, and we spent a lot of social time just hanging out, while we waited for each day’s inevitable Havana-specific delays to resolve themselves. In this way, I was able to analyse how the material geography and spatial imaginaries of Old Havana were produced through the performance of contrasting discourses of Old Havana as a ‘living museum’ as imagined by revered city historian Eusebio Leal (Rodirguez Alomá and Fornet, 2011), and Old Havana as ‘home’ as imagined by the muchachos.

We used the Plaza de San Francisco as our central meeting point; it is the gateway for tourists arriving to the city by cruise ship, so I had many opportunities to observe the muchachos sharing the public space with tourists. What I report in this article is a combination of the experiences that the muchachos shared with me through the formal research activities mentioned above and in our informal social time, and the experiences that I witnessed and shared in during our time together.

### The muchachos’ missing ‘backstage’

**Figure 2.**
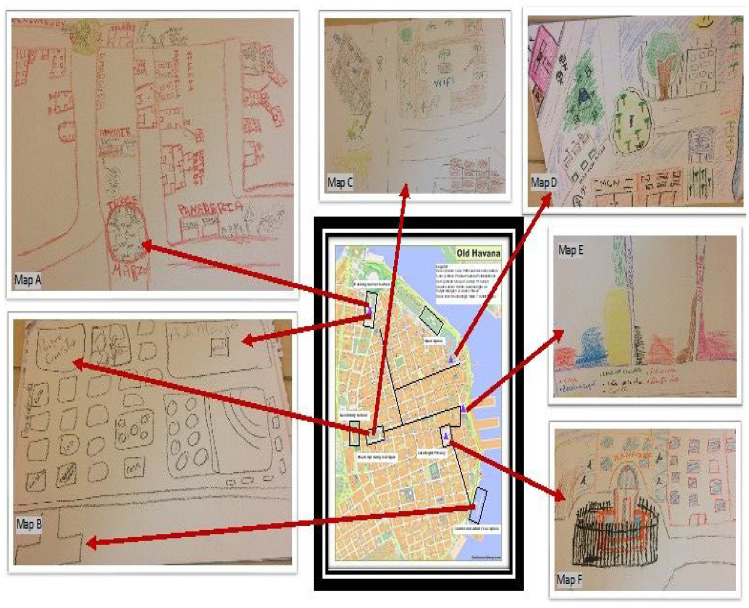
Some of the muchachos’ neighbourhood maps contextualised within the composite map of their use of space in Old Havana. *Source*: Muchachos.

Virtually all of the social activities that the muchachos reported took place in public spaces; meeting friends to play football (mostly the boys), to run errands together (mostly the girls), to sit and chat, to meet new people or try to ‘run into’ romantic interests (both) all happened in streets, parks and squares (as highlighted on the maps in Figure 2 which illustrate where they spent their free time). It is well-documented that low-income youth disproportionately bear the burden of the negative effects of globalisation on urban contexts (Gunter, 2010; Katz, 2004). The restoration of Old Havana is no different, as it physically deprives young people of ‘backstage’ (Goffman, 1978) spaces around the community. The spatial practices (Lefebvre, 1991) that produce and reproduce space in Old Havana as a site of heritage tourism directly and indirectly dispossess young people of the places needed for these developmentally significant peer relationships: the kind of ‘hanging out’ teens need to do away from the gaze of authority figures such as parents, neighbours, teachers and the police, through the enclosure, sanitisation and temporary appropriation of public spaces in which youth previously hung out.

The developmental need for adolescents to withdraw from adult surveillance and engage in peer-only identity formation activities is often discussed in terms of young people’s need for private space in which they can control who observes their behaviour as they experiment with different aspects of their identity (Abbott-Chapman and Robertson, 2009). Identity scholars engage Goffman’s (1978) dramaturgical metaphor of ‘front’ and ‘backstage’ regions to describe how young people rehearse and protect their role performances before exposing them to the scrutiny of ‘outsiders’, or those with authority. The definition of ‘front’ and ‘backstage’ regions is tied more closely to audience control (whose gaze can reach us) than it is to built form (if there are walls around us) (McCarthy, 2005), and often young people find the kind of privacy needed for ‘backstage’ behaviour in public places (Aitken, 2005; Tani, 2015; Valentine, 2017).

Extreme overcrowding in Old Havana forces multiple generations to live together in small homes. Most of the muchachos lived in *solares*, with living space that measured 5 m^2^ and sleeping space in an improvised loft above. Overcrowding denies the muchachos of private space at home. Due to the market reliance of the OHCH’s model, its need to prioritise the restoration of buildings for income-generating businesses over social housing means that the rehabilitation of Old Havana is slow to alleviate overcrowding (Peters, 2001; Scarpaci, 2000, 2012) and the muchachos are forced to live their social lives in public spaces, exposing them to more surveillance and social control than youth with access to private social spaces.

The muchachos used the term *descargar* (to unload) to describe what they did when they met with their friends. This term highlights the psychological value of these interactions, in which they *unload* their thoughts and feelings and process them together. Sometimes confusingly, they also used the term *descargar* to refer to ‘making out’ and some of them talked about romantic or sexual relationships of varying degrees of formality. Having no private space for peer relationships in adolescence is a challenge because public places do not always offer the ‘backstage’ space required for peer-only activities. Both types of relationship unfolded almost exclusively in public spaces: squares, parks, curbs and the floating dock for platonic interactions, and rooftops, stairwells, alleyways and dark corners for romantic or sexual interactions. The process of restoration that is prioritising commodifiable spaces over social housing for local families is also dispossessing the muchachos of the public spaces on which they rely.

## Processes of dispossession and displacement

We identified three main processes through which the dispossession of public space and the displacement of the muchachos took place in Old Havana in ways that were different from other parts of the city: the commodification of public space through de facto enclosure, the sanitisation of public space through the spatial practices of a variety of interested actors and the temporary appropriation of de jure public spaces by tourists or OHCH restoration activities.

### Enclosure

Spaces that were previously usufruct housing, small businesses serving locals or informal commons are being remade into bars, restaurants or rental accommodation for tourists. As these new spaces are produced, old spaces are destroyed and are not replaced. The muchachos often referenced important ‘backstage’ spaces of which they had been dispossessed through this process of enclosure. These places held symbolic significance in the muchachos’ imaginations of their community and practical significance in their activities, hence their frequent appearance in stories, neighbourhood tours and mapping. As the informal commons of empty lots, building ruins, outdoor stairways, parkettes and curbs disappears from the muchachos’ community maps, they are forced into the formal public spaces of parks and squares which come with their own set of constraints for people like them (see Figure 2).

The changing nature of public space in Old Havana was highlighted by Alejandro (16), who put into question the absolute value of the term ‘public’, suggesting that even de jure public spaces are becoming de facto spaces for consumption:

[Joanna] Given your resources, which spaces *are* accessible to you?

[Alejandro] I have access to … practically … given my resources, I have access to very few places, very, very, very few, really just to public spaces, the *most* public spaces are the only ones I have access to, where I can go without having to pay, without having to use money, because everything here is very, very, very expensive, expensive meaning … where would I *get* that money from?

**Figure 3.**
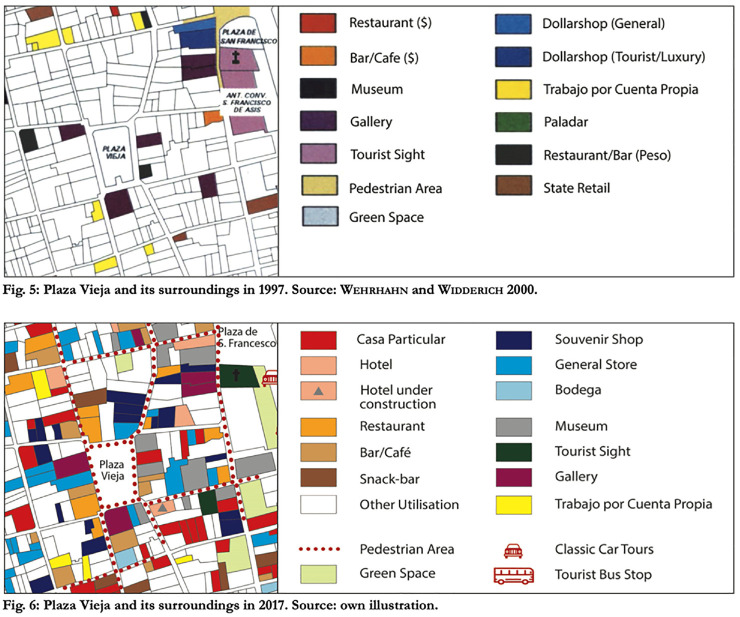
Changes in land use around Plaza Vieja, 1997 to 2017. *Source*: Völkening et al. (2019).

Völkening et al. (2019) trace the shift in the use of space in Old Havana between 1994 and 2017, noting a quintupling in private businesses serving tourists (easily distinguished by their exclusive use of foreign currency), and a 90% reduction in state-run institutions serving locals. For example, Figure 3 maps the changes in the use of space around the Plaza Vieja in that time, a process that has intensified even more with recent policy changes regarding small businesses. Businesses with frontage on squares or pedestrianised streets effectively enclose much of that space for patios or merchandise displays.

The reimagining of Old Havana as a space of tourist consumption has led to a social restructuring that leaves the muchachos out. Their inability to consume in foreign currency restricts their access to newly created spaces while shifting the responsibility for their exclusion to the muchachos themselves, justified through economic rationality. Local people are not legally excluded from such spaces, but they cannot afford the services or goods they provide. The muchachos identified this economic exclusion when we asked them to tell us which spaces in the community were ‘for them’ and which spaces were ‘not for them’. Consistently, they responded that spaces that were ‘not for them’ were bars, restaurants, shops and hotels designed to serve tourists, and they identified affordability as the determining criteria. All of the muchachos spoke to this economic exclusion, but Alejandro (16) articulated the neoliberal paradox clearly: ‘tengo acceso, pero no tengo para hacerlo accesible’ (‘I have access, but I don’t have the means to make it accessible’). He highlighted the consumption function of these spaces:

[Alejandro] I can go in, I can go in and sit down, but once you’re in there you have to do something! That’s what makes it inaccessible

[Javier] You don’t have money to consume …

[Alejandro] There is no money to consume!

The messages of this exclusion were internalised by the muchachos in different ways, although one thing their responses had in common was the feeling of being valued less than foreigners. The muchachos identified the high prices and inaccessibility of these spaces as one of their top priorities for change in their neighbourhood and discussed how their exclusion felt. When she said ‘No one goes in there’ of a new tourist bar on her block, Maria (14) was making clear the us/them distinction that has emerged between tourists and locals. ‘No one [of *us*] goes in there’, she meant, because ‘that’s made for *yumas* [foreigners], not for us’. ‘They charge US$1.50 for a coffee, that’s an insult, that’s not success, it’s just rude’, Sara (13) said. While they expressed frustration at being excluded from these spaces, they also expressed desire to be valued in the way foreigners were in these establishments: ‘There’s just more quality for *yumas*. They treat them well, they serve them nicely, I’d like that’, Miguel (14) said. ‘I wish we had the quality of products they gave *yumas* in places that I could go’, said Juan (16).

The revitalisation of Old Havana is producing the early stages of what Christopherson (1994: 410) calls ‘the fortress city’; one that centres consumption and neglects ‘the city as a home, a place of cultural expression and a political venue’. This transformation has impacts on how the muchachos understand their place in society and how they imagine their futures, linking their value closely to their ability to consume.

### Sanitisation

The unique ways in which processes of neoliberal urbanism specifically impact marginalised youth tend to play out in public spaces (Kennelly and Watt, 2013), which are a key terrain for the urban entrepreneurial activities of the OHCH (Oficina del Historiador de La Habana, 2021). McCann (2009: 121) identifies two ways in which the physical environment of a city is reshaped through processes that flank city marketing:

(1) the building or renovation of elements of the built environment to enhance or correspond to a marketing goal and (2) the policing, cleaning, and managing of urban public spaces with the express intention of making them correspond to a carefully constructed brand and, thus, to make them feel safe and welcoming for those who the [marketer] sees as its most valuable clients.

The spatial practices that produce spaces for heritage tourism are essential to the city marketing mandate of the OHCH to project a safe and welcoming city and, as a result, often unsettle the muchachos’ sense of belonging in these public spaces. The OHCH custodians charged with enforcing the new spatial imaginary in Old Havana’s restored colonial squares are often at odds with local youth. The following is an excerpt from an individual interview I was conducting with one muchacho as we sat on the edge of one such square, an activity we considered unremarkable, but one that did not escape the surveillance of the custodian.

Guided Neighbourhood Tour – 4 pm, 30 October 2017: Plaza Vieja, Old Havana

Joanna: What’s the biggest downside of living in this neighbourhood?

Luis (17): Well for my age it’s not so … it doesn’t affect me as much really … but for the younger ones it’s very difficult to use this space to play, you’ve got this big square, but you can’t play fútbol here, not here, not in Plaza de las Palomas [local name for Plaza San Francisco], I have to go really far away to play fútbol.

Joanna: If you tried to play fútbol here the police would stop you?

Luis: Yes, they don’t let us play, they tell you you can’t play here.

OHCH Custodian who has been listening in interrupts interview: They don’t let you play, no! This is a square.

Luis: But you can’t pl –

OHCH Custodian: [interrupting]: This is a square, not a park.

Luis: That’s what I’m sa –

OHCH Custodian: [interrupting]: No.

Joanna: Fútbol is not allowed in the plaza?

Luis: That’s what I’m trying to s –

OHCH Custodian: [interrupting]: The police don’t stop you; this is a square, it’s for walking, you can’t ride a bicycle here, you can’t play here, what if you hit somebody, a tourist or a child? It’s not some unreasonable thing, you just can’t play here.

Joanna: It’s not a place designed to play in?

OHCH Custodian: A square is a square and a park is a park.

Joanna: Which is the nearest park to here where you’re allowed to play fútbol?

OHCH Custodian: I don’t know. There are plenty. … [lists a few that we know do also not allow fútbol]. Listen, I’m the custodian of this square!

As the above exchange illustrates, the active management of public space is essential to marketing the reimagined city in ways that often do not suit the muchachos. They recognised the value of public space, and Alejandro (16) highlighted its political character:

In the square, any kind of person has access, anyone can come and have the same enjoyment of it that everyone else has, but in a bar, maybe we all have access, but we don’t all have the same enjoyment because in a bar you don’t buy the same drink as that other guy, you can’t afford the same bottles he can, there’s an important difference between a bar and the square.

The muchachos are not valued clients of the OHCH’s project, and its spatial practices of public space management disrupt their place identity and with it their sense of belonging in the few spaces they can still access in their neighbourhood.

The ‘backstage’ character of public space was further compromised by the surveillance and policing of public space that is considered essential to the production of a safe and inviting space for foreign consumers (Coleman, 2004). While surveillance and policing are pervasive throughout Cuba’s public and private spaces, behaviours of concern in most places are limited to those that are dangerously illegal or potentially counter-revolutionary, while in the public spaces of Old Havana, behaviours that might threaten the sense of safety of tourists are closely monitored and quickly addressed. Victoria (16) articulated the panopticist nature of public space and its impact on the ‘backstage’ suitability of such places:

When you’re using a public space, you have to be guided by a certain discipline, one that suits everyone, for the good of everyone, but in a private space you create your own discipline, in your house, you know ‘this is my house and I can do what I want!’

The enforcement of such discipline in Old Havana is highly internalised by the muchachos, who have grown up surrounded by surveillance cameras, plain-clothed police and a culture that incentivises informing on possible dissidents to achieve political capital (Colomer, 2000). Their expressions of their identity are conditioned by what they feel safe saying and doing under such scrutiny.

One afternoon, as Alejandro (17) and I stood outside the Convent of San Francisco preparing for our outdoor theatre performance, we were approached by two police officers who restrained him and held him up against a wall nearby. They accused him of being a *jinetero* and told him to leave me alone. It was only when I explained that we worked together and asked them to let him go that they released him, apologised to me and walked away without another word to him. The message was clear, my experience as a White Canadian woman *was* worth more to them than his. Overly authoritative and intimidating police interactions are not uncommon, especially for young Black men living in Old Havana, who are often perceived as threatening to tourists. Since the priority of local police is to maintain public spaces that are safe and welcoming for tourists, the experience of the muchachos and their peers is not their concern.

Beyond formal processes of sanitisation, businesses engage in spatial practices that make the muchachos feel unwelcome around the public spaces they have enclosed as patios or merchandise displays. Hanging out near these spaces would often get the muchachos accused of *jineterismo*, or otherwise disrupting clients’ sense of safety, even when these spaces were on the literal doorsteps of their homes. Such interactions unsettled the muchachos’ sense of belonging and impacted their identity work by forcing them to reconcile these negative views of themselves.

### Temporary appropriation

The ways in which people make use of space determines the meanings those spaces have and impacts the ways in which others may use that space; the appropriation of space is a basic socio-spatial process and has been the focus of much urban theory, especially when it comes to public spaces (Purcell, 2002; Lara-Hernandez et al., 2020). Jover and Díaz-Parra’s (2020) work exploring overtourism in the historic centres of European cities warns that failing to differentiate between the collective appropriation of space by a community and the temporary ‘consumption’ of space by visitors can result in the erasure of place alienation. The tension between these two competing uses of public space is palpable in many of the muchachos’ experiences.

The muchachos’ dispossession of public spaces through their remaking as tourist-serving sites takes place both physically and symbolically, through formal and informal activities. At times, public spaces that were important to the muchachos were not available to them because they were being occupied by tourists; large tour groups spreading out around a square to take photographs would ask the muchachos to move out of their shot, clusters of tourists would sit on the few viable benches, walls or steps available, tourists would shelter from the sun or rain in the few covered spots where muchachos spent their social time. The temporary appropriation of these spaces by tourists who wanted to experience the local flavour of Old Havana displaced the vessels of said flavour, forcing them to move out of the area to find new places to hang out.

**Figure 4.**
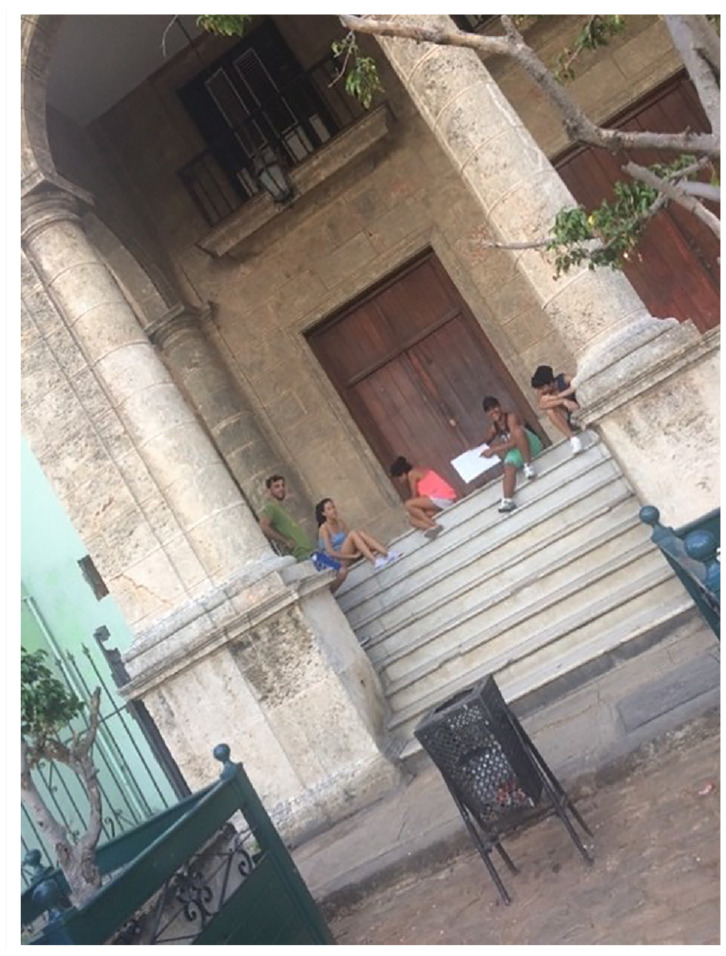
The muchachos making neighbourhood maps in our outdoor office, the stairs of the former CUPET office, which were closed off when the building underwent OHCH-managed restoration to become a five-star hotel. Note the elegant, frequently emptied rubbish receptacle in the foreground. (Photo: Author).

The process of rehabilitation dispossesses the muchachos of public spaces during construction, as several of the public spaces that the muchachos use get blocked off as a result of nearby construction work. The closure of the stairs of the CUPET building used by our group as an outdoor office (see Figure 4) drove the muchachos to sit on the stone pedestal around the Galician monument on the other side of the Plaza. The area around the monument was soon closed off as well, due to construction on an adjacent building. With the closure of these two spaces, and the constant use of the plaza’s fountain for tourist photography, the muchachos were fully dispossessed of the Plaza de San Francisco and were forced to find a new part of the city to make their meeting point.

I observed the muchachos largely ignoring and avoiding tourists, reluctantly and resentfully giving up space to them when they felt the gaze of foreign tour operators, local tourist-serving entrepreneurs or the police. Not all interactions between the muchachos and tourists were so subtle, however. Plaza Vieja is the largest of the colonial squares in Old Havana; it is surrounded by restaurants, art galleries, museums and a school. One day, as the muchachos and I were standing in the square talking, a tour operator stretched out his arms and pushed our group out of the way without addressing any of the muchachos. He then waved his group into the spot where we had been standing. The muchachos, angry and humiliated, shuffled back reluctantly and then suggested we relocate. When I asked them about it, they grumbled that they were used to it, and it wasn’t worth causing trouble. Such an interaction, and those I recount below, are examples of how ‘space is produced through performances, building material geographies from the embodiment of discourse’ (Watkins, 2015: 517), in this case of who is entitled to use the square.

The entitlement of tourists to the idealised spatial imaginary of Old Havana as a ‘living museum’ is not limited to its space. The Yoruba religion and culture of Afro-Cubans is heavily represented in Old Havana and is often advertised as one of the ‘exotic’ elements that gives the neighbourhood its ‘colour’. The Yoruba consecration process involves several ceremonies during which the *iyawo* (person being consecrated) dresses in all white, uses special jewellery, covers their head and must observe various restrictions around physical contact and photography.

One of the muchachos underwent this process during the time of our study. One day, he and I were inside Casa de Carmen Montilla gallery when a tour group entered. The tour operator encouraged them to surround us, pointing at the *iyawo*, touching his clothes and necklaces, speaking in German to the tourists who photographed us. Neither the tour operator nor the tourists made eye contact with or spoke to the *iyawo*. When they left, the *iyawo* commented that they treated him worse than the contents of the art gallery, noting that no one would just start touching the paintings and that someone would stop them if they did.

The entitlement of the tour operator and tourists to the culture of Old Havana was both symbolic and physical. Reflecting a process that Collins (2011) labels the enclosure of human practice, the tourists felt they could consume the *iyawo*’s personal religious experience as if it were a museum exhibit, and touch his body, clothes and jewellery without making human contact with him. The *iyawo* was just the kind of local flavour tourists are sold in promotional materials that ‘aid in the naturalisation of spatial imaginaries, moving ideas, arguments, and consequential “otherings” across space and time’ (Watkins, 2015: 511). He was key to their ‘authentic’ experience of Old Havana, and their objectification of him is the kind of behaviour that makes the muchachos feel like they are less valued than tourists in their own communities.

## Young peoples’ changing place(s)

The enclosure, sanitisation and temporary appropriation of public spaces restricted the muchachos’ ability to use them, let alone contest or resist disrespectful or aggressive treatment by foreigners or police in the public spaces they call home. One muchacho pointed out how the management of public space is about creating a pro-tourist space, not about maintaining public order, as behaviour is tolerated differently depending on whose it is. The Plaza Vieja, where the OHCH custodian diligently manages the behaviour of the muchachos, is described by Alejandro (17) as ‘the place with the beer garden and all the drunken tourists’. This discrepancy highlights the failure of the ‘security’ or ‘public safety’ discourse invoked by the custodian to account for ‘the differences in classification and treatment of young people who perform similar types of “deviant” behaviour but occupy different racial and class positions’ (Hickey-Moody, 2013: 57).

The contrast between the use of public space by local teens and rowdy tourists pouring out of the beer garden highlights the importance of consumption in the reimagined Old Havana. The economic restructuring of the community means that, in addition to bearing the brunt of public space management, local people are also excluded from the new tourist-serving private spaces that are created. Decisions about who gets to be where and under what conditions communicate messages about value and worth that play a role in the muchachos’ identity work.

**Figure 5.**
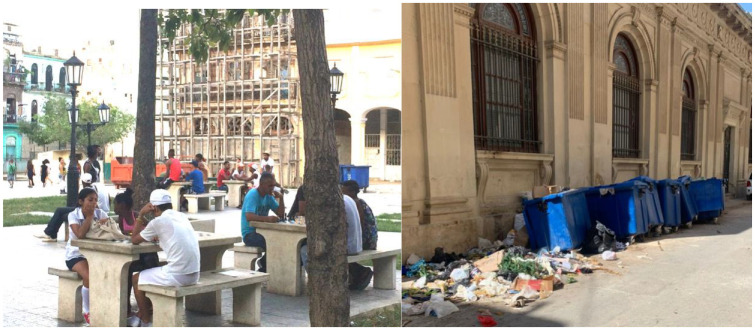
Left: Young people from around Old Havana fill Parque de Cristo, which is the only square in the neighbourhood which has not yet been formally restored by the OHCH. It is known as the most ‘caliente’ or dangerous of the neighbourhood’s public spaces. (Photo: Author); Right: Rubbish containers overflow into Old Havana’s unprioritised streets. (Photo: L Varona).

For example, the physical and social changes in the built environment of the places prioritised for rehabilitation, such as the four restored squares and the streets that connect them, have impacts on the physical and social spaces that have not been rehabilitated. Echoing historical patterns of disinvestment in Old Havana, the OHCH is forced to concentrate investment in places of heritage-tourism significance, which starves places of significance to the local community of resources. In their drawings, the muchachos often illustrated this concentration of resources through the juxtaposition of exquisitely maintained green spaces, squares and buildings with derelict children’s playgrounds, kids playing fútbol around jagged broken pavers and locals perched carefully on benches with just one splintered board left. Muchachos who lived close to El Parque de Cristo (see Figure 5) also noted that the sanitisation of other plazas pushed locals into this one unrestored square, increasing the density of its use and increasing the rates of conflict and insecurity there. During our neighbourhood tours, many noted that while diligent custodians monitor the carefully maintained squares, rubbish containers overflow onto the pavement and streets just metres away.

This new spatial–symbolic relationship in which people like the muchachos are relegated to a position of inferior importance and worthiness communicates messages about their value that impact how the muchachos see themselves. The muchachos reported that being cast as a threat, unworthy of quality goods and services, unwelcome, unwanted or commodified in the places they call home contributes to a sense of frustration, abandonment and resentment. The need to constantly modify their behaviour to suit the demands of the tourism industry spills over into their imagined futures. While most of the muchachos identified career ambitions in professions such as engineering, teaching, art and medicine, many of them recognised the comparatively low financial payoffs they offer in the new Cuban economic context. Several of them mentioned that if they failed to achieve their career ambitions, they could ‘just find work in a bar if they had to’. All of the muchachos pursued post-secondary education but all but two left school to work in the service industry, reflecting the conflict between what the muchachos felt was personal success and how they could achieve it, and the changing society around them that values people working in the service industry more highly than doctors or teachers.

While ‘othering’, enacted through the enclosure, sanitisation and temporary appropriation of public spaces, for example, is a well-documented and highly criticised product of globalisation and neoliberal urbanism (McCann, 2009; Sassen, 2007; Wacquant, 2008), the participatory and inclusive rhetoric of the OHCH and foreign enthusiasm for Cuba’s first post-Revolutionary foray into capitalist markets have shielded the revitalisation process of Old Havana from such critique. Watkins (2015) labels this a ‘spatial transformation imaginary’, the means through which a process is naturalised or made invisible or inevitable to facilitate its execution. The spatial transformation imaginary depicting Old Havana’s restructuring as universally beneficial and economically necessary has been naturalised through a centralised planning approach that involves infrequent and tokenistic public engagement, limiting opportunities for alternative discourses to emerge and circulate.

The hard edges of touristification and gentrification have been softened by the inclusion of residents and local life as artefacts in a ‘living museum’, and the language of restoration, which suggests a return to something that has been there all along, or that belongs in Old Havana, as opposed to the introduction of something new. The ideological and spatial practices that flank this transformation secure its apparent inevitability, redefining what the future of the community looks like in ways that condition local youths’ identities. While the OHCH’s careful approach to the touristification of Old Havana has resisted the kind of housing-led displacement that is common in the gentrification of historic urban centres, unquestioned practices of enclosure, sanitisation and temporary appropriation mean that the current course of the neighbourhood’s transformation threatens to reinforce the economic and social exclusion of people like the muchachos, whose place identities are essential to their current wellbeing and future development.

## Notes

1. Reymer Sanchez, Junior Costa, Javier Pino, Dania Perez and Rachel Perera from the Department of Recreation at the Universidad de Ciencias de la Cultura Física y el Deporte Manuel Fajardo, in Havana, to whom I will be eternally indebted.

2. The muchachos’ ages are reported throughout based on their age at the time of each quote.

3. Available at https://joannakocsis.wixsite.com/jovenhabanafilm.
